# GWRM: An R Package for Identifying Sources of Variation in Overdispersed Count Data

**DOI:** 10.1371/journal.pone.0167570

**Published:** 2016-12-09

**Authors:** Silverio Vílchez-López, Antonio José Sáez-Castillo, María José Olmo-Jiménez

**Affiliations:** 1 IES Las Fuentezuelas, Jaén, Spain; 2 Department of Statistics and Operations Research, University of Jaén, Jaén, Spain; University of New South Wales, AUSTRALIA

## Abstract

Understanding why a random variable is actually random has been in the core of Statistics from its beginnings. The generalized Waring regression model for count data explains that inherent variability is given by three possible sources: randomness, liability and proneness. The model extends the negative binomial regression model and it is not included in the family of generalized linear models. In order to avoid that shortcoming, we developed the GWRM R package for fitting, describing and validating the model. The version we introduce in this communication provides a new design of the modelling function as well as new methods operating on the associated fitted model objects, so that the new software integrates easily into the computational toolbox for modelling count data in R. The release of a plug-in in order to use the package from the interface R Commander tries to contribute to the spreading of the model among non-advanced users. We illustrate the usage and the possibilities of the software with two examples from the fields of health and sport.

## Introduction

The apparently chaotic behaviour of any random variable reveals that, in general, we do not know how and why data vary. In the case of count data, the Poisson distribution [1] provides a very simple answer: the random nature of the variable is due to pure chance. Anyway, most of the observed variables show a more complex structure of variability, since the Poisson assumptions (independent counts and constant rate of occurrence) are quite restrictive in real applications. That is the origin of mixed Poisson distributions, which allows the occurrence rate to vary among different cases; in the end, they assume there are two sources of variability in the counting process, pure chance and differences between individuals. The negative binomial (*NB*) distribution [2] would probably be the most well-known Poisson mixture, although many others have been adequately studied in recent years [3].

In this context, the Univariate Generalized Waring Distribution (*UGWD*) [4] allows for identifying a new possible source of variability. It may be considered as a mixture of a *NB* distribution, in such a way that when a count variable follows a *UGWD*, it may be inferred that the variable is affected by three possible sources of variation: pure chance (or randomness), different exposures to the risk in the counting process (liability) and differences only due to individual characteristics (proneness). The terms randomness, liability and proneness come from Irwin [4]. Unfortunately, the main drawback of this capability to split the sources of the variability is that liability and proneness cannot actually be distinguished without some extra information or some subjective judgement about the problem under consideration [5].

Precisely, in a regression framework this extra information is given by a set of covariates. And that is the ground of the Generalized Waring Regression Model (*GWRM*) [6]. If we have a sample consisting of *n* cases for which we know the result of the counting process, *y*_*i*_, and a series of common covariates, **x**_**i**_, for *i* = 1, …, *n*, a *GWRM* fitting this dataset would permit the inference that the counts are affected by a) pure chance (randomness); b) variability due to different exposures to risk related to the existent covariates (liability); and c) variability due exclusively to individual differences not related to the covariates (proneness). In this way the components liability and proneness are perfectly distinguished.

Based on this feasibility in the variability explanation, the *GWRM* has been employed already in different contexts. In relation to errors in geographical datasets, the *GWRM* was fitted to the number of errors in cells of 1 × 1 *km*^2^ on the Topographic Map of Andalusia (Spain) with some covariates [7]: the empirical relationship established by the model identified the significant covariates and, moreover, showed that for cells having less than 5 errors, most of the variability corresponded to unknown external factors (liability), whereas when the number of errors rose, the greater part of the variability was due to unknown internal characteristics of each cell (proneness). In accident analysis, two applications in the modelling of crash data in segments of roads were carried out [8], concluding that proneness represented the over-dispersion due to between-segments variation in their internal probability to cause accidents with the same values of the covariates, while liability was related to the over-dispersion caused by missing external covariates which would affect them; such information is valuable because it can help transportation safety professionals to better control the variance found in traffic crashes by implementing more cost-effective safety countermeasures without having to conduct a full identification of hazardous sites. Analysing the determinants of scientific research production at individual level by means of three different indicators—based on the number of publications and/or citations—, considered as response variables, the corresponding distributions were observed as highly skewed and displaying an excess of zero-valued observations [9]; the goodness-of-fit of several Poisson mixture regression models, including the *GWRM*, was compared by assuming an extensive set of explanatory variables, resulting that this model showed a good performance in terms of Akaike Information Criterion (AIC) values. The *GWRM* has been also considered as a form of mixed *NB* distribution to model the excess of variability in relation to the Poisson distribution (over-dispersion) [10]. Finally, it could also be mentioned a Bayesian version of the *GWRM* which permits an estimation of the posterior distribution of the proneness of footballers in relation to their ability to score goals [11].

We think these studies are a demonstration of the strength of the *GWRM* in the context of regression in count data. Thus, we want to contribute by means of this work to its spread with the detailed description of an easy-to-use software for non-advanced users, the GWRM package [12] of R [13] and its R Commander plug-in [14, 15], which have been designed as a set of statistical functions to fit, validate and describe a *GWRM*. The fitting function included is based on the maximum-likelihood principle and it is implemented via different numerical methods. This fitted model can be described by means of common inferential analysis: in particular, the significance of the covariates is evaluated by the Wald test, although the likelihood ratio test (LRT) is also possible. Precisely the LRT, but also the AIC and the BIC (Bayesian Information Criterion), may be employed to carry out a stepwise procedure (forward, backward or in both directions) to select the covariates of the model. The analysis of the residuals, to assess the adequacy of the model to data, has been implemented by a bootstrap envelope for deviance, Pearson and response residuals; that the actual distribution of the residuals is unknown must be taken into account, since assumptions to be considered as normal or Poisson residuals are violated. Finally, a specific method has been implemented to describe the sources of variation to the fitted models: it provides a partition of the variance of each combination of the covariates in three terms, corresponding to randomness, liability and proneness.

The paper is structured as follows. The second section reviews the genesis and the properties of the *GWRM*, comparing it with the Poisson (*PRM*) and negative binomial regression (*NBRM*) models. In the third section, the functions of the GWRM package and the main features of the R Commander plug-in are described, and two examples to illustrate the use of the package are included, one in the health field and another in the area of sport. In the final section, the paper concludes with a summary of the main characteristics of the package implemented.

## The generalized Waring regression model

Let *Y* be the response variable of a count model so that *Y*|**x** follows a *Poisson*(λ_**x**_) where ${\text{x}}^{\prime }=\left(\begin{array}{cccc} 1 & x_{1} & \cdots & x_{p} \end{array}\right)$ is the vector of covariates. Considering the effect that the covariates have on the mean in a log-linear scale, that is

$$\begin{array}{c} \lambda_{\text{x}}=e^{{\text{x}}^{\prime }\beta }, \end{array}$$

where $\beta^{\prime }=\left(\begin{array}{cccc} \beta_{0} & \beta_{1} & \cdots & \beta_{k} \end{array}\right)$ is the parameter vector, the *PRM* arises. This model is characterized by the property of equidispersion, that is, *Var*(*Y*|**x**) = *E*(*Y*|**x**) and, as we mentioned in the introduction, represents total randomness: once we know the covariate values of a case, its response value is due only to pure chance. Nevertheless, in most of the applications the variability of data exceeds the mean, which is known as overdispersion. The usual way to cope with overdispersion is to consider that the ratio of occurrences λ_**x**_ is not the same for all the observations with the same values of the covariates, but it varies from one observation to another following a random model, which leads to their definition as mixed Poisson models. The most common solution is to assume that λ_*x*_ ∼ *Gamma*(*a*_**x**_, *v*_**x**_). In this case, *Y*|**x** follows a *NB*(*a*_**x**_, *p*_**x**_), with *p*_**x**_ = 1/(1 + *v*_**x**_). The regression model obtained is known as *NBRM* and the conditional mean is given by

$$\begin{array}{c} E\left(Y|\text{x}\right)=E\left(E\left(Y|\text{x},\lambda_{\text{x}}\right)\right)=E\left(\lambda_{\text{x}}\right)=\mu_{\text{x}}=a_{\text{x}}v_{\text{x}}. \end{array}$$

Then, if *μ*_**x**_ = *e*^**x**′***β***^ and *v*_**x**_ does not depend on the covariates (*v*_**x**_ = *v*), the *NegbinI* model appears [16]. In this model, the variance-mean rate is constant since *Var*(*Y*|**x**) = (1 + *v*)*μ*_**x**_. On the other hand, if *a*_**x**_ does not depend on the covariates (*a*_**x**_ = *a*), the *NegbinII* model appears, with a linear variance-mean rate, $Var\left(Y|\text{x}\right)=\mu_{\text{x}}\left(1+\frac{1}{a}\mu_{\text{x}}\right)$.

Focusing on the *NegbinI* model

$$\begin{array}{c} Var\left(Y|\text{x},v\right)=E\left(Var\left(Y|\lambda_{\text{x}}\right)\right)+Var\left(E\left(Y|\lambda_{\text{x}}\right)\right)=E\left(\lambda_{\text{x}}\right)+Var\left(\lambda_{\text{x}}\right)=a_{\text{x}}v+a_{\text{x}}v^{2}. \end{array}$$

The first term, *E*(λ_**x**_) = *a*_**x**_ *v* = *μ*_**x**_, is the variability due to the randomness inherent in the Poisson distribution, while the second term *Var*(λ_**x**_) = *a*_**x**_ *v*^2^ = *vμ*_**x**_ represents the heterogeneity across individuals which causes overdispersion. It may be highlighted that both sources of variation, *Poisson*(λ_**x**_) and *Gamma*(*a*_**x**_, *v*), change in terms of the covariate values.

If in the *NegbinI* model we consider *v* ∼ *BetaII*(*ρ*, *k*), then the response variable has a univariate generalized Waring distribution (*UGWD*) with p.m.f.

$$\begin{array}{c} f\left(y|\text{x}\right)=\frac{\Gamma \left(a_{\text{x}}+\rho \right)\Gamma \left(k+\rho \right)}{\Gamma \left(a_{\text{x}}+k+\rho \right)\Gamma \left(\rho \right)}\frac{{\left(a_{\text{x}}\right)}_{y}{\left(k\right)}_{y}}{{\left(a_{\text{x}}+k+\rho \right)}_{y}}\frac{1}{y!},\;y=0,1,2,..., \end{array}$$

where *a*_**x**_, *k*, *ρ* > 0 and (*α*)_*r*_ = Γ(*α* + *r*)/Γ(*α*) for *α* > 0 is a Pochhammer symbol. The mean is given by

$$\begin{array}{c} E\left(Y|\text{x}\right)=\mu_{\text{x}}=a_{\text{x}}/\left(\rho -1\right), \end{array}$$

so *ρ* > 1 must be imposed in order to guarantee its existence. Again, considering the effect of the covariates on the mean as *μ*_**x**_ = *e*^**x**′***β***^, the *GWRM* arises and

$$\begin{array}{c} a_{\text{x}}=\frac{\mu_{\text{x}}\left(\rho -1\right)}{k}. \end{array}$$

For further details of this regression model see [6].

The variance of the model is now given by

$$\begin{array}{ccc} Var(Y|\text{x}) & = & E(Var(Y|\text{x},v))+Var(E(Y|\text{x},v))\\ & = & E\left(E\left(\lambda_{\text{x}}\right)\right)+E\left(Var\left(\lambda_{\text{x}}\right)\right)+Var\left(E\left(Y|\text{x},v\right)\right), \end{array}$$

so the introduction of a new random component in the *NegbinI* model allows an interpretation of the variability in terms of three sources of variation:

1. The first term,
  $$\begin{array}{c} E\left(E\left(\lambda_{\text{x}}\right)\right)=E\left(a_{\text{x}}\right)=a_{\text{x}}\frac{k}{\rho -1}=\mu_{\text{x}}, \end{array}$$
  represents the variability due to randomness, which comes form the underlying Poisson model.
2. The second one,
  $$\begin{array}{c} E\left(Var\left(\lambda_{\text{x}}\right)\right)=E\left(a_{\text{x}}v^{2}\right)=a_{\text{x}}\frac{k(k+1)}{\left(\rho -1)(\rho -2\right)}=\frac{k+1}{\rho -2}\mu_{\text{x}}, \end{array}$$
  represents the average of the variability in the different exposures to risk, λ_**x**_, given by the different values of the covariates, which comes from the gamma model. The parameter *ρ* must be greater than 2 in order to guarantee the existence of the variance. Since covariates may be seen as external factors, we will consider this term as the variability due to liability. The more differences the gamma model establishes among the individuals for each λ_**x**_, the higher the importance of this component will be.
3. The third one,
  $$\begin{array}{c} Var\left(E\left(Y|\text{x},v\right)\right)=Var\left(a_{\text{x}}v\right)=a_{\text{x}}^{2}\frac{k(k+\rho -1)}{{\left(\rho -1\right)}^{2}\left(\rho -2\right)}=\mu_{\text{x}}^{2}\frac{k+\rho -1}{k(\rho -2)}, \end{array}$$
  is the variability due to the introduction of an individual component for each individual from the beta model; since that individual value does not depend on the covariates, we can consider it as due to internal factors or proneness.

In relation to the limiting cases of the *GWRM*, it may be proved [6] that if *k*, *ρ* → ∞ with the same order of convergence, the *GWRM* tends to a *NegbinI* model, while if *ρ* → ∞ and *μ*_**x**_/*k* is bounded, the *GWRM* tends to a *NegbinII* model.

Finally, the possibility of the infinite variance effect (when *ρ* < 2), suggests a heavy-tailed behaviour; in relation to that, it is easy to prove

$$\begin{array}{c} \lim_{y\to \infty }\frac{f(y|\text{x})}{y^{-(\rho +1)}} \end{array}$$

is a positive constant, so, in fact, the *UGWD* is a power-law.

## Using the GWRM package

### Overview

The R package GWRM has been written to fit the *GWRM*. The entire package has been recently rewritten from its initial version to conform with the usual requirements of R regression packages. It provides a rich interface using standard functions and methods for object-oriented computations. In some aspects, the implementation of the package has been inspired by the glm() and lm() functions of the stats package.

The source code is available on the Comprehensive R Archive Network, CRAN, repository (http://cran.r-project.org/web/packages/GWRM) with all the information about its functions and parameters in the package help. It can be installed and loaded by typing the following commands in R:

> install.packages(“GWRM”)

> library(GWRM)

Since the package is open-source, it is also available in GitHub (https://github.com/ujaen-statistics/GWRM) where updates and comments can be submitted.

The GWRM package provides the modelling function

gw(formula, data, weights, k = NULL, subset, na.action, kstart = 1, rostart = 2, betastart = NULL, offset, control = list(…), method = NULL, hessian = TRUE, model = TRUE, x = FALSE, y = TRUE, …)

which returns an object of class gw. This function is used to fit a *GRWM*, specified by giving a symbolic description of the linear predictor. With respect to the initial version of the package, the potential optimizers are now interfaced in a more functional approach and starting values and other convergence criteria can be supplied. Specifically, the default fitting method initially uses non-linear minimization (nlm) and Nelder-Mead optimization (optim) to fit a model which is then re-fitted by “L-BFGS-B” (optim). In this way, standard error (SE) estimates for all the model parameters are provided. The optimization methods nlm and Nelder-Mead are also possible values for the argument method, but they do not provide SE estimates for the parameters *k* and *ρ*. In that case, the method estimates the parameters *ρ*_0_ and *k*_0_ and their SE, where

$$\begin{array}{c} k=e^{k_{0}},\;\rho =1+e^{\rho_{0}}. \end{array}$$

These restrictions are necessary to guarantee the existence of the mean of the model.

The results provided by the function gw() together with their description are listed in the help (see help(gw)).

The package also has a print() and summary() method. The generic and standard functions coef(), logLik(), AIC(), BIC(), predict(), residuals(), add1(), drop1() and step(), available in R regression packages, can be applied to a gw object. The possibility of using methods and standard functions has improved the initial version of the package.

The function residuals() returns residuals of type pearson (default), deviance and response. Deviance residuals are defined as

$$\begin{array}{c} d_{i}=2\left[\ln f\left(y_{i}|y_{i}\right)-\ln f\left({\hat{\mu }}_{i}|y_{i}\right)\right] \end{array}$$

so that $D=\sum_{i=1}^{n}d_{i}$ is the value of the deviance statistic. In the new version of the package we have included the option of drawing a normal plot with a simulated envelope of the residuals. This plot is a useful technique for analysing the residuals [17, 18]. Generally it has been treated as an informal check of model fit: if the fitted model is correct, the plotted points are all likely to fall within the boundaries of the envelope, so the existence of some points outside the envelope will be a sign of lack of accuracy. A graphical method is more informative than a single numerical test of fit since the shape of the plot may indicate where and of what type is the lack of accuracy and, what is more, it could help to detect outliers. By default the number of simulations for the construction of the envelope is 19, so there is a chance of 1 in 20 (a 5%) that the largest absolute residual from the original data set falls outside the simulated envelope, when the fitted model is appropriate.

The package provides a parallel interface to the function residuals() that includes the arguments parallel (by default TRUE) and ncores (by default 2).

Moreover, the package contains the function partvar() which splits the variance of a *GWRM* into three components. The first component of this decomposition represents the variability due to randomness, the second refers to liability and the third to proneness. The output shows the absolute value of each component as well as their respective proportions.

### Examples

For illustrative purposes we provide here two examples. The first one is an application in the health field to propose a *GWRM* for the number of visits to doctor in relation to some explicative variables. The second one refers to the number of goals scored by footballers in the first division of the Spanish league in the last ten seasons. The R code for reproducing these examples as well as the corresponding output are in the Supporting Information (S1 and S2 Files, respectively).

#### Number of visits to doctor

We use the set of data badhealth available in the R package COUNT. They were obtained from the German health survey for the year 1998 only and consist of 1127 observations on the following 3 variables:

- numvisit: Number of visits to doctor during 1998.
- badh: 1 if the patient claims to be in bad health or 0 if is not in bad health.
- age: the age of patient (from 20 to 60 years old).

Firstly, we fit the model considering numvisit as the response variable and only including the independent term

> library(GWRM)

> library(COUNT)

> data(badhealth)

> badhealth.gw0 <- gw(numvisit ˜ 1, data = badhealth)

The command summary(badhealth.gw0) shows the coefficient estimates, their standard errors and the associated partial Wald tests (statistics and *p*–values). The R output also contains the degrees of freedom and the estimation method finally used along with the code of convergence.

Call: gw(formula = numvisit ˜ 1, data = badhealth)

Coefficients:

Estimate  Std. Error   z value   Pr(>|z|)

(Intercept)  8.554e-01   4.419e-02  1.936e+01   1.844e-83

Fit:

log-likelihood    AIC  BIC  df

-2283      4572  4587  1124

betaII:

par    Estimate   Std. Error

k     1.773977   0.857405

ro    4.178057   0.886486

Degrees of Freedom: Total (i.e. Null); 1124 Residual

Code of convergence: 0

Method: L-BFGS-B

Next, the function step() allows us to select the best formula based on the *AIC* or *BIC* (with the argument k = log(n)):

> badhealth.finalgw   <- step(badhealth.gw0, scope = ˜ badh + age,

+ data = badhealth)

The resulting fitted regression model is given by:

> summary(badhealth.finalgw)

Call: gw(formula = numvisit ˜ badh, data = badhealth)

Coefficients:

Estimate  Std. Error   z value    Pr(>|z|)

(Intercept)   6.581e-01   4.143e-02  1.589e+01   8.032e-57

badh     1.162e+00   1.069e-01  1.087e+01   1.572e-27

Fit:

log-likelihood   AIC   BIC   df

-2228      4465  4485  1123

betaII:

par    Estimate  Std. Error

k     1.567578  0.3305424

ro     6.852336  1.8424138

Degrees of Freedom: Total (i.e. Null); 1123 Residual

Code of convergence: 0

Method: L-BFGS-B

Now the *GWRM* fit is compared with the *PRM* and *NBRM* fits:

> badhealth.pois   <- glm(numvisit ˜ badh + age, family = poisson,

+ data = badhealth)

> summary(badhealth.pois)

Call:

glm(formula = numvisit ˜ badh + age, family = poisson, data = badhealth)

Deviance Residuals:

Min    1Q   Median    3Q   Max

-3.6653   -1.9186   -0.6789  0.6292   10.0684

Coefficients:

Estimate   Std. Error   z value   Pr(>|z|)

(Intercept)  0.447022  0.071428  6.258     3.89e-10  ***

badh    1.108331  0.046169  24.006      <2e-16  ***

age     0.005822  0.001822  3.195    0.0014 **

---

Signif. codes: 0 ?***? 0.001 ?**? 0.01 ?*? 0.05 ?.? 0.1 ? ? 1

(Dispersion parameter for poisson family taken to be 1)

Null deviance: 4020.3 on 1126 degrees of freedom

Residual deviance: 3465.3 on 1124 degrees of freedom

AIC: 5638.6

Number of Fisher Scoring iterations: 5

> library(MASS)

> badhealth.nb <- glm.nb(numvisit ˜ badh + age, data = badhealth)

> summary(badhealth.nb)

Call:

glm.nb(formula = numvisit ˜ badh + age, data = badhealth,

init.theta = 0.9974812528, link = log)

Deviance Residuals:

Min    1Q    Median    3Q   Max

-2.0304     -1.4361   -0.4152  0.3180  3.9516

Coefficients:

Estimate Std.   Error   z value   Pr(>|z|)

(Intercept)  0.404116    0.130847  3.088  0.00201  **

badh    1.107342    0.111603  9.922  < 2e-16  ***

age     0.006952    0.003397  2.047  0.04070  *

---

Signif. codes: 0 ?***? 0.001 ?**? 0.01 ?*? 0.05 ?.? 0.1 ? ? 1

(Dispersion parameter for Negative Binomial(0.9975) family taken to be 1)

Null deviance: 1355.7 on 1126 degrees of freedom

Residual deviance: 1217.7 on 1124 degrees of freedom

AIC: 4475.3

Number of Fisher Scoring iterations: 1

Theta: 0.9975

Std. Err.: 0.0693

2 x log-likelihood: -4467.2850

It is remarkable that, in contrast with the *GWRM* model, the *PRM* and *NBRM* ones include age as a significant covariate at 5% significante level. Anyway, using the *AIC*, the best fit is that provided by the *GWRM*, even when it has one less covariate.

The prediction for each combination of the covariates (badh = 0 and badh = 1) using the *GWRM* fit are 1.931035 and 6.173427, respectively. The first one, for example, is obtained by:

> badh.0 <- subset(badhealth, badh == 0)

> predictions.finalgw.0 <- predict(badhealth.finalgw, newdata = badh.0)

> predictions.finalgw.0[1,]

Therefore, a patient who claims to be in bad health visits the doctor on average between 6 and 7 times a year; whereas a patient who claims not to be in bad health visits the doctor on average twice a year.

Typing the command partvar(badhealth.finalgw) the absolute value and the proportion of the variance components for each combination of the covariates, that is, randomness, liability and proneness, are obtained. The proportion of these components for the first individual (since there are no other covariates, all the individuals have the same value of these variance components) is obtained by:

> partvar.finalgw.0   <- partvar(badhealth.finalgw, newdata = badh.0)

> partvar.finalgw.0$Prop.Variance[1,]

Randomness Liability Proneness

1 0.2930119 0.1550451 0.551943

> badh.1   <- subset(badhealth, badh == 1)

> partvar.finalgw.1   <- partvar(badhealth.finalgw, newdata = badh.1)

> partvar.finalgw.1$Prop.Variance[1,]

Randomness Liability Proneness

10 0.1324292 0.07007398 0.7974968

Results indicate that those patients who claim to be in bad health have less than half of variability due to randomness and liability, and greater variability due to proneness (around 25% higher) than those who claim not to be in bad health.

Trying to illustrate the role of liability we have obtained the partition of the variance for the *GWRM* fitted to this data but including the covariate age. For the sake of brevity the code and the output are included as supplementary material. It can be observed that variability due to liability decreases when the covariate age is included in the model, that is, there are less differences among individual risks when the age is present, whereas variability due to randomness hardly varies. Certainly, this reduction is not high, but it should be remembered that this variable is not significant.

**Fig 1:**
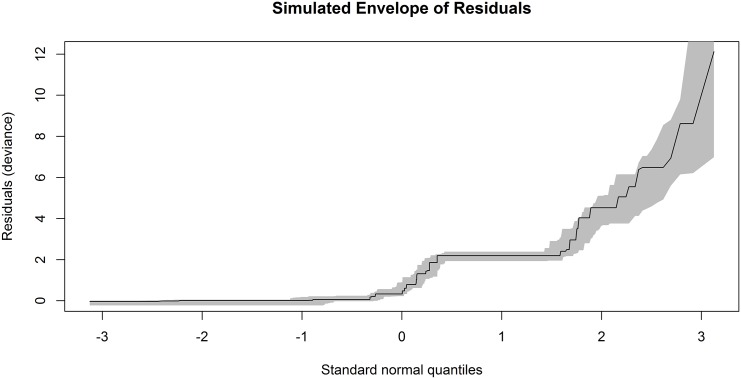
Simulated envelope of the residuals of the *GWRM* fit to badhealth data. Plot of the deviance residuals against the order statistics of the normal distribution from the *GWRM* fitted to the number of visits to doctor during 1998.

Finally, the QQ-plot with the simulated envelope for the deviance residuals is drawn (Fig 1). Taking into account that the sample size is high (*n* = 1127), some points could fall outside the boundaries of the envelope by pure chance using the 19 simulations by default. So, we have considered 99 simulations. This figure is provided by the command residuals(badhealth.finalgw, type = “deviance”, envelope = TRUE, rep = 99). As the line of the residual points matches the shape of the simulated envelope and moreover all of them lie inside the simulated envelope, we can conclude that there is no evidence against the adequacy of the fitted model.

#### Number of goals scored

We consider data about the number of goals scored by the footballers in the first division of the Spanish league. Data have been collected from the web page http://www.bdfutbol.com from 2003/2004 to 2013/2014 seasons and are available in the Supporting Information (S1 Dataset). The population is composed of 4082 footballers, excluding goalkeepers and those who have not played entire matches.

These count data show a clear overdispersion that can be due to a set of external factors that significantly influence the risk of scoring a goal, but also to internal factors, related to the footballer’s goal-scoring ability and intelligence.

With this example we try to illustrate how the *GWRM* is able to capture these sources of variability in comparison with other usual regression models for overdispersed count data, such as the *NBRM*. Then, we have modelled the response variable *number of goals scored by a footballer* in terms of the covariate *position in the field*, with three levels: forward, midfielder and defender. It has been coded by two dummy variables, with defender as the reference category. We have also included the variable *number of entire matches played*, with values from 1 to 38, as an offset, that is, an exposure variable. The use of this offset instead of a more natural exposure variable such as the exact number of minutes played is exclusively due to the illustrative purpose of the example, because it contributes to introduce extra-variability, which increases the potential advantage of the *GWRM* model in comparison with *PRM* and *NBRM*.

We have fitted the model in the last ten seasons in order to analyse the evolution of the partition of the variance by means of the following code:

> Spain <- read.table(“Spain.txt”)

> library(GWRM)

> library(MASS)

> gw.fits <- list()

> nb.fits <- list()

> for (i in 1:10){

gw.fits[[i]] <- gw(Goals ˜ Position + offset(log(Matches)),

+ data = Spain[Spain$Season == levels(Spain$Season)[i], ])

nb.fits[[i]] <- glm.nb(Goals ˜ Position + offset(log(Matches)),

+ data = Spain[Spain$Season == levels(Spain$Season)[i], ])

}

Again, using the *AIC*, these fits are better than the *PRM* and *NBRM* fits. Specifically, the improvement of the *AIC* along the ten fitted seasons is given by

>— sapply(gw.fits, function(data) AIC(data))

+ sapply(nb.fits, function(data) AIC(data))

[1] 0.6983898 1.6834744 14.8675845 14.8601620 1.7582653 12.8896949

[7] 9.1630974 5.2755611 11.7468735 4.1297644

**Fig 2:**
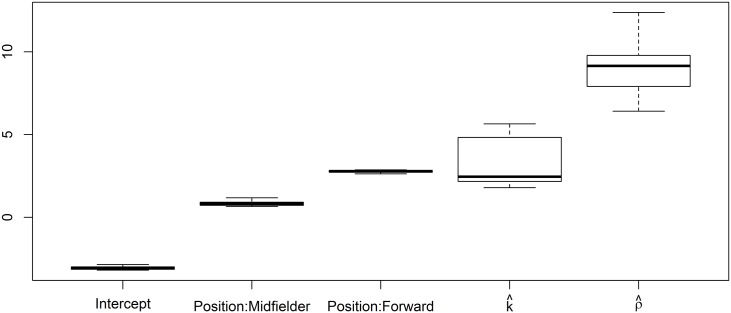
*GWRM* parameter estimates. Each box plot summarizes the set of ten estimates of each parameter obtained from the fits of the ten season datasets.

Fig 2 shows the box plots for the estimates of *β*_0_, *β*_1_, *β*_2_, *k* and *ρ* along the ten seasons. It can be observed that the regression coefficient estimates are quite similar in all the seasons. This figure is generated by the code

> boxplot(t(sapply(gw.fits, function(l) l$coefficients))[, c(1,3,2,4,5)],

+ sd = TRUE, names = c(“Intercept”, “Position:Midfielder”,

+ “Position:Forward”, expression(hat(k)), expression(hat(rho))),

+ eyes = FALSE)

**Fig 3:**
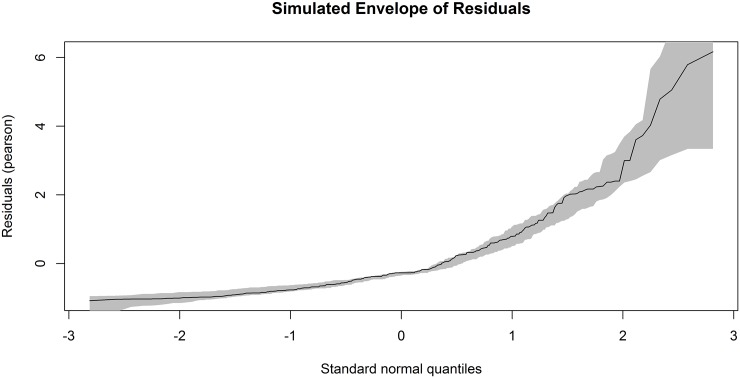
Simulated envelope of the residuals of the *GWRM* fit to goals data. Plot of the Pearson residuals against the order statistics of the normal distribution from the *GWRM* fitted to the goals scored by the footballers in the first division of the Spanish league in the 2004/2005 season.

Now, the adecuacy of the *GWRM* fits is checked using the QQ-plot with the simulated envelope for the Pearson residuals. As an example, Fig 3 represents the simulated envelope with 99 simulated samples for the 2004/2005 season. It has been obtained with the command res <- residuals(gw.fits[[1]], envelope = TRUE, rep = 99). Although the line of the residual points matches the shape of the simulated envelope, there are some of them (8 points) which lie outside, so we can conclude that there is a certain lack of fit in the fitted model of that season.

**Fig 4:**
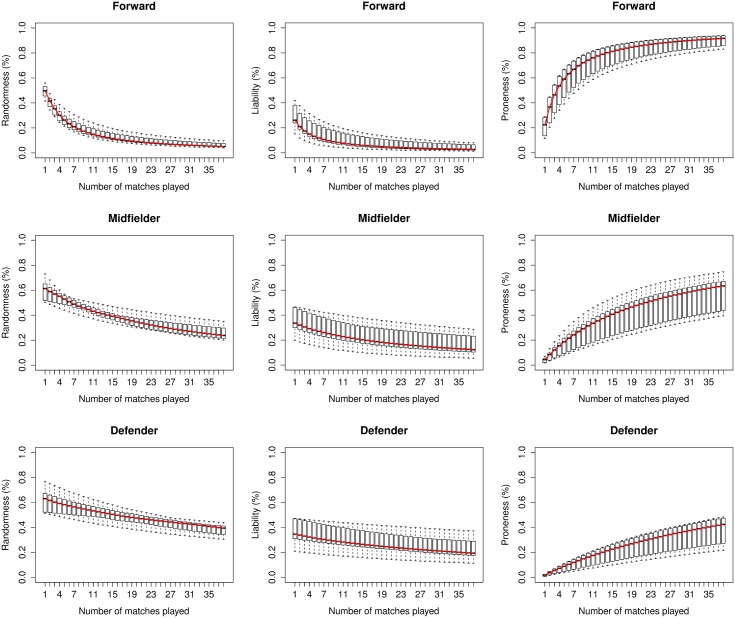
Proportion of the variance components for each position. Each boxplot summarizes the ten values of the variance partition component obtained from the fits of the ten season datasets.

Fig 4 shows the proportion of the variance related to randomness, liability and proneness for each position, in terms of the number of matches played. In turn, for each value of the number of matches played there is a box plot where all the seasons are represented. Also, the medians are joined with a red line to show the evolution of the respective proportion of the variance. The code that generates this figure is provided as supplementary material and it is not included here for the sake of brevity.

From this figure we can deduce that:

- In general, the variability in the number of goals scored due to randomness and liability decreases as the number of matches increases in all the seasons, whereas the variability due to proneness increases. So, we can deduce that increasing the number of matches emphasizes the role of individual characteristics (proneness) as a cause of the differences between players in relation to the number of goals scored, whereas pure chance (randomness) and other external factors which establish differences in the scoring goal risk within each position in the pitch (liability) become less relevant.
- Taking into account the footballer’s position in the pitch, forwards have greater variability due to proneness than midfielders and defenders. Moreover, the variability due to randomness is greater for defenders, followed by midfielders and forwards. This shows two interesting aspects. Firstly, differences between forwards, with regard to goals scored, are more related to their goal-scoring intuition than in the case of midfielders; the same happens with midfielders in comparison with defenders. Secondly, variability between defenders, related to the number of goals scored, is mainly due to pure chance (randomness) instead of to their goal-scoring intuition (proneness) or other external factors associated with the position in the pitch (liability).

## A plug-in for R Commander

**Fig 5:**
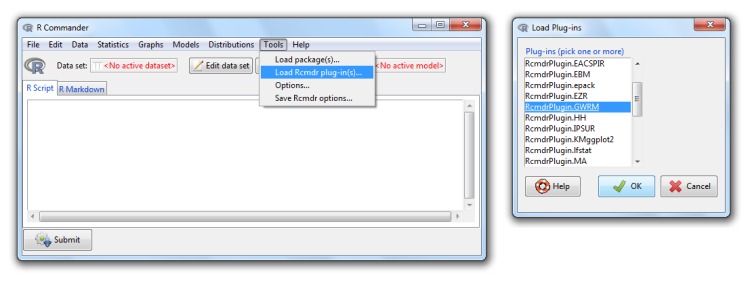
Installing the GWRM plugin for R Commander.

In order to expand the use of the GWRM package to non-advanced users, we have developed a plugin for R Commander [15], a basic-statistics GUI for R. The plugin is a package called RcmdrPlugin.GWRM that can be downloaded using install.packages() and that can be loaded only once R Commander has been invoked through the load plugin menu option (see Fig 5). The code of this package is also available in GitHub (https://github.com/ujaen-statistics/RcmdrPlugin.GWRM).

**Fig 6:**
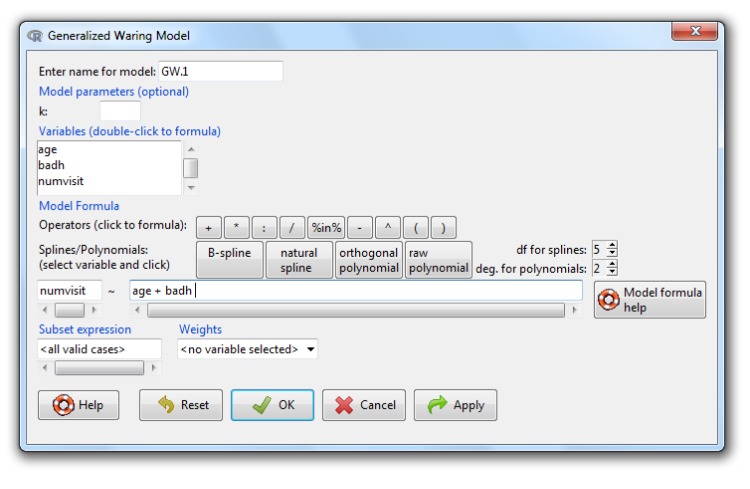
The *GWRM* dialog box.

**Fig 7:**
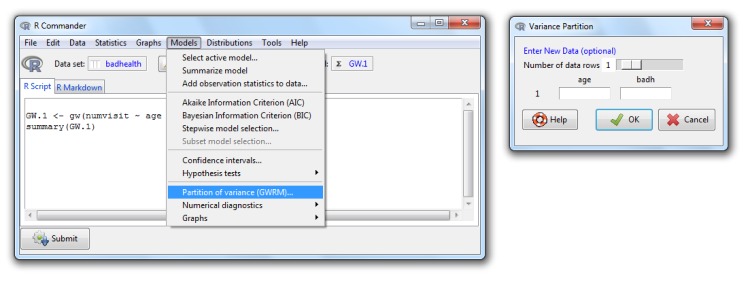
The *GWRM* option *partition of variance* and its dialog box.

**Fig 8:**
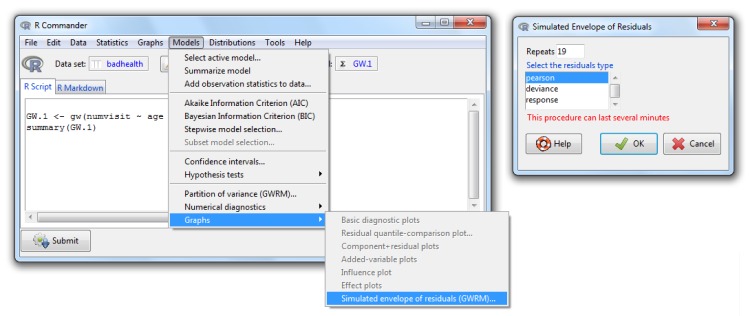
The *GWRM* option *simulated envelope of residuals* and its dialog box.

Selecting *Statistics*→*Fit models*→*Generalized Waring model (GWRM)* from the main menu brings up the dialog box shown in Fig 6, which shares a common general structure with that of the *Linear Model*. Therefore, the use of this dialog box is similar to the linear model except the box labelled *Model parameters*, in which a fixed value for the parameter *k* can be specificied; if it is not supplied, the *k* estimate is computed. Operations on the active model may be selected from the *Models* menu. The specific ones for the *GWRM* are:

- *Partition of variance*, which provides the components of the partition of the variance for the whole data set (*Number of data rows* = 0) or for specified values of the model covariates (*Number of data rows* = 1, 2, …) (see Fig 7).
- *Graphs*→*Simulated envelope of residuals (GWRM)*, which shows a QQ-plot with the simulated envelope for the Pearson, deviance or response residuals (you can select one the three types) with 19 simulated samples (Fig 8). Again, you can use the whole data set (*Number of data rows* = 0) or specify a new one (*Number of data rows* = 1, 2, …).

## Conclusions

The GWRM package has been designed for fitting, describing and validating the generalized Waring regression model for overdispersed count data, which is not included in the family of generalized linear models. As well as estimating the parameters of the model, the package includes tools that allow for splitting the data variability into three components: randomness, liability and proneness. Moreover, a simulated envelope of the residuals can be drawn in order to check the validity of the fitted model. The package has been inspired by the glm and lm functions of the stats package, so its use is very similar. Nevertheless, to facilitate the use of this package to non-advanced R users, a plug-in for the GUI R Commander has also been implemented.

## Supporting Information

S1 Dataset Data about the number of goals scored by the footballers in the first division of the Spanish league. (TXT)

S1 File Code presented in the manuscript in order to reproduce the examples with R. (R)

S2 File Output from the above-mentioned code. (TXT)

## References

[1] Poisson SD. Probabilité des jugements en matière criminelle et en matière civile, précédées des règles générales du calcul des probabilitiés. Paris, France: Bachelier 1837.

[2] Greenwood M, Yule GU. An inquiry into the nature of frequency distributions representative of multiple happenings with particular reference to the occurrence of multiple attacks of disease or of repeated accidents. Journal of the Royal Statistical Society. 1920; 255–279. 10.2307/2341080

[3] Karlis D, Xekalaki E. Mixed poisson distributions. International Statistical Review. 2005; 73(1): 35–58. 10.1111/j.1751-5823.2005.tb00250.x

[4] Irwin JO. The generalized Waring distribution applied to accident theory. Journal of the Royal Statistical Society Series A (General). 1968; 205–225. 10.2307/2343842

[5] Xekalaki E. The multivariate generalized Waring distribution. Communications in Statistics-Theory and Methods. 1986; 15(3): 1047–1064. 10.1080/03610928608829168

[6] Rodríguez-Avi J, Conde-Sánchez A, Sáez-Castillo AJ, Olmo-Jiménez MJ, Martínez-Rodríguez AM. A generalized Waring regression model for count data. Computational Statistics and Data Analysis. 2009; 53(10): 3717–3725. 10.1016/j.csda.2009.03.013

[7] Ariza-López FJ, Rodríguez-Avi J. Estimating the count of completeness errors in geographic data sets by means of a generalized Waring regression model. International Journal of Geographical Information Science. 2015; 29(8): 1394–1418. 10.1080/13658816.2015.1010536

[8] Peng Y, Lord D, Zou Y. Applying the Generalized Waring model for investigating sources of variance in motor vehicle crash analysis. Accident Analysis and Prevention. 2014; 73: 20–26. 10.1016/j.aap.2014.07.031 25173723

[9] Baccini A, Barabesi L, Cioni M, Pisani C. Crossing the hurdle: the determinants of individual scientific performance. Scientometrics. 2014; 101(3): 2035–2062. 10.1007/s11192-014-1395-3

[10] Faddy MJ, Smith DM. Analysis of count data with covariate dependence in both mean and variance. Journal of Applied Statistics. 2011; 38(12): 2683–2694. 10.1080/02664763.2011.567250

[11] Sáez Castillo A, Rodríguez Avi J, Pérez Sánchez JM. Expected number of goals depending on intrinsic and extrinsic factors of a football player. An application to professional Spanish football league. European Journal of Sport Science. 2013; 13(2): 127–138. 10.1080/17461391.2011.589473

[12] Sáez-Castillo AJ, Vílchez-López S, Olmo-Jiménez MJ, Rodríguez-Avi J, Conde-Sánchez A, Martínez-Rodríguez AM. GWRM: Generalized Waring Regression Model for Count Data; 2016. GWRM R package version 2.0.2. Available from http://CRAN.R-project.org/package=GWRM

[13] R Core Team. R: A Language and Environment for Statistical Computing. Vienna, Austria; 2015. Available from: http://www.R-project.org/

[14] Sáez-Castillo AJ, Vílchez-López S, Olmo-Jiménez MJ. RcmdrPlugin.GWRM: R Commander Plug-In for Fitting Generalized Waring Regression Models; 2016. RcmdrPlugin.GWRM R package version 1.0.1. Available from: http://CRAN.R-project.org/package=RcmdrPlugin.GWRM

[15] Fox J. The R Commander: A Basic-Statistics Graphical User Interface to R. Journal of Statistical Sofware. 2005; 14(9): 1–42.

[16] Cameron AC, Trivedi PK. Regression Analysis of Count Data. Cambridge University Press; 2013.

[17] Atkinson AC. Plots, Transformations and Regression: An Introduction to Graphical Methods of Diagnostic Regression Analysis. Oxford Science Publications; 1985.

[18] Vieira AMC, Hinde JP, Demetrio CGB. Zero-inflated proportion data models applied to a biological control assay. Journal of Applied Statistics. 2000; 27(3): 373–389. 10.1080/02664760021673

